# Tuneable conductivity at extreme electric fields in ZnO tetrapod-silicone composites for high-voltage power cable insulation

**DOI:** 10.1038/s41598-022-09966-4

**Published:** 2022-04-11

**Authors:** Helena Greijer, Nicola Mirotta, Emanuele Treossi, Filippo Valorosi, Fabian Schütt, Leonard Siebert, Yogendra Kumar Mishra, Rainer Adelung, Vincenzo Palermo, Henrik Hillborg

**Affiliations:** 1 Hitachi Energy Research, 72178 Västerås, Sweden; 2 ISOF – Istituto per la Sintesi Organica e la Fotoreattivita, CNR Area Della Ricerca di Bologna, Via P. Gobetti 101, 40129 Bologna, Italy; 3 Institute for Materials Science, Functional Nanomaterials, Kiel University, Kaiser Str. 2, 24143 Kiel, Germany; 4 Smart Materials, NanoSYD, Mads Clausen Institute, University of Southern Denmark, Alsion 2, 6400 Sønderborg, Denmark; 5 Department of Industrial and Materials Science, Chalmers University of Technology, 41258 Göteborg, Sweden

**Keywords:** Electronic properties and materials, Electronic devices, Organic-inorganic nanostructures, Polymers

## Abstract

Resistive Field Grading Materials (RFGM) are used in critical regions in the electrical insulation system of high-voltage direct-current cable systems. Here, we describe a novel type of RFGM, based on a percolated network of zinc oxide (ZnO) tetrapods in a rubber matrix. The electrical conductivity of the composite increases by a factor of 10^8^ for electric fields > 1 kV mm^−1^, as a result of the highly anisotropic shape of the tetrapods and their significant bandgap (3.37 eV). We demonstrate that charge transport at fields < 1 kV mm^−1^ is dominated by thermally activated hopping of charge carriers across spatially, as well as energetically, localized states at the ZnO–polymer interface. At higher electric fields (> 1 kV mm^−1^) band transport in the semiconductive tetrapods triggers a large increase in conductivity. These geometrically enhanced ZnO semiconductors outperform standard additives such as SiC particles and ZnO micro varistors, providing a new class of additives to achieve variable conductivity in high-voltage cable system applications.

## Introduction

Global energy demand is continuously increasing, requiring efficient transmission and distribution of large amounts of energy over long distances, which drives rapid development of polymeric insulation materials used in electrical power applications. One recent example uses extruded high-voltage direct-current (HVDC) cable systems, operating at voltages up to 525 kV^1^. However, further developments in materials to control electrical stress distribution in HVDC cable insulation systems are required^2^. Electrical stress distribution is especially high in critical regions such as interfaces and triple points, which dramatically increases the probability of electrical breakdowns, resulting in failure of the power transmission line. Issues involving such critical regions can be mitigated by using resistive field-grading materials (RFGMs). Such materials are insulating at the nominal voltage level, but become more conductive at higher electric fields, thereby redistributing the electric field. Thus, the electrical conductivity increases dramatically once a threshold value of the electric field (*E*_*th*_) is reached^3–7^. The local E-field is thus reduced, along with the risk of electrical breakdown. Therefore, RFGMs reduce the maximum E-field stress at critical locations within the cable insulation. The conductivity under normal operation is the low field conductivity (*σ*_0_) and above *E*_*th*_ the conductivity rapidly increases thereby decreasing the local E-field. A non-linearity coefficient (*α*) describes how quickly this increase takes place. In general, stress grading improves with increasing nonlinearity, but α values well above 20–30 do not add much further benefit^5^. Since Joule heating may be significant during these over-voltage events, this aspect needs to be considered in the thermal design of any given insulation system, to avoid overheating of the RFGM. However, given that such events are of short duration, significant effects on energy loss are unlikely. In addition, it is beneficial to minimize the low-field conductivity under normal operation. This reduces energy loss during continuous use of the insulation system. RFGMs used in HVDC cables usually consist of percolated (> 25 vol%) composites based on block-like, inorganic semi-conductive particles, such as silicon carbide, typically with linear dimensions of 10 μm, in polymers such as ethylene propylene diene monomer (EPDM) or silicone rubber^3,4,7^. Another approach is based on polymer composites filled with a percolated (> 25 vol%) network of spherical ZnO micro varistor particles, with linear dimensions typically in the range of 30–100 μm^5,8^. The benefit of these types of RFGMs is their isotropic conductivity. However, the high filling factors of semiconducting material needed for high quality state-of-the-art RFGMs also often bring disadvantages. The high filler levels impose limitations due to poor mechanical properties and higher weight^9^.

In polymer nano-composites electrical percolation can be obtained at much lower loadings (even below 1%) by using high aspect ratio additives with anisotropic shape, such as 1-dimensional nanotubes or 2-dimensional nanosheets^10^. Shapes including pointed spikes or sharp edges with high curvature will enhance the electric field locally at the tips, thus favouring the generation of free charge carriers at high electric fields. The use of anisotropic additives for RFGM has already been successfully demonstrated using thermally reduced graphene oxide nanosheets or BaTiO_3_ nanofibers in silicone rubber^9,11^. One main difficulty in using anisotropic fillers for RFGM is that the conductivity will have a complex, non-linear, non-monotonic dependence on the orientation of the fillers^12,13^, as found in a specific case of polymers filled with 1-D carbon nanotubes^14^. The conductivity of the composite can increase by a factor of 10–1000 on moving from completely random orientations to certain degrees of alignment. However, when going above a critical degree of anisotropy, the higher alignment of the 1-D fillers will decrease the probability of two of them being in close enough contact; thus, the electrical conductivity will decrease again. This complex behaviour will also depend upon filler content and aspect ratio, especially at lower filler fractions. Controlling such alignment in real materials, such as injection moulded composites where the local conductivity will vary with the flow fields generated during moulding, is difficult^12^, and this has so far hindered the use of 1-D fillers in RFGM. Here, the issue of processing and alignment control is not addressed, but circumvented by using a geometrical workaround, as detailed below.

A simple way to obtain pointed spikes and high local electric fields with any orientation is to use tetrapod-shaped semiconductors. The angle between the arms of the tetrapod is 109.5°; thus, there will be always an arm oriented nearly parallel to an applied electric field, with an angle less or equal to 54.75°. Bearing in mind that one of the most commonly used materials for RFGM (ZnO) can also be grown as tetrapods, and in large quantities, using various physical and chemical methods, in particular by flame transport synthesis. Such ZnO tetrapods are promising for applications in electronics, composites, coatings, biomedical materials, energy harvesting, cosmetics, pharmaceuticals, etc^15^. A ZnO tetrapod basically consists of four 1-dimensional ZnO nanorods, hence having all the physical and chemical features of the 1-D nanorods, with a direct bandgap of 3.37 eV^15^ which is also ideally suited for RFGM applications. Thus, to exploit the fortunate combination of shape, bandgap and processability of ZnO tetrapods we added them to a silicone rubber matrix, commonly used for HVDC cable insulation. The results obtained were compared with similar tests previously done on commercial, state-of-the-art composites, and analysed by extracting the activation energy of the process, to unravel the charge transport mechanisms in the composite.

## Experimental

**Figure 1:**
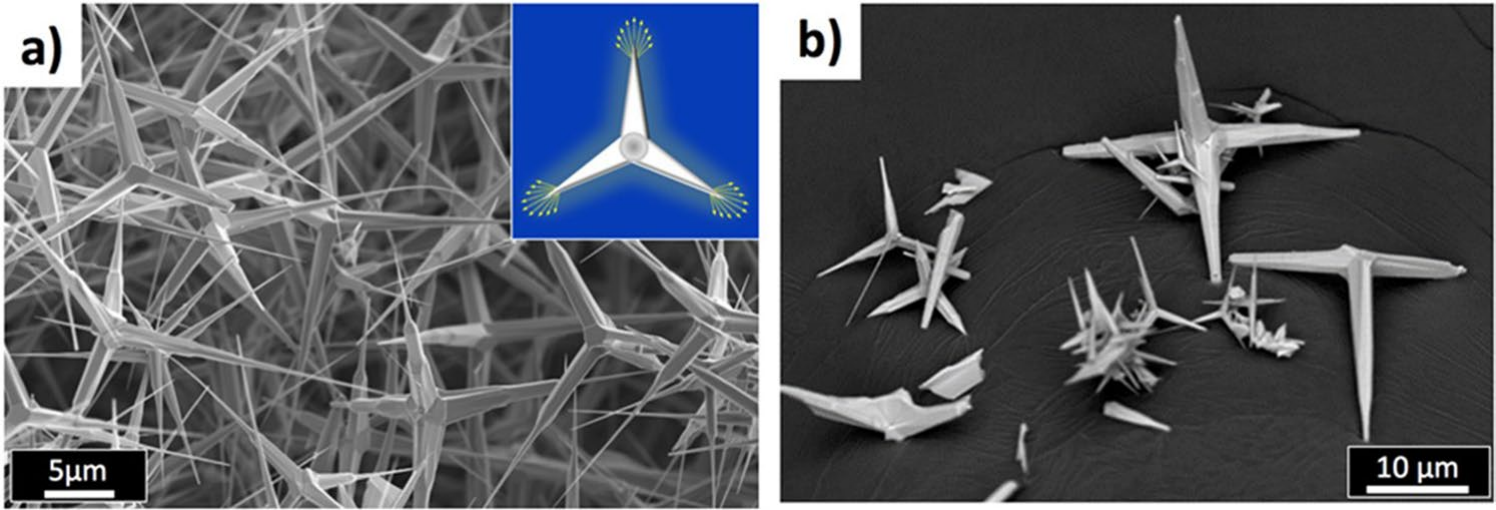
(**a**) SEM image of ZnO tetrapods grown by flame annealing. Inset: cartoon showing how the tips of the tetrapod can enhance the electric field for any given orientation of the field. (**b**) SEM image of the ZnO–silicone composite after dissolving part of the polymer in xylene, uncovering the tetrapods present in it.

ZnO tetrapods (Fig. 1a) were obtained using flame transport synthesis^16^. Zn precursor particles are exposed to a flame which creates a laminar or partially turbulent upstream, fostering the direct transformation of the metal into ZnO tetrapods. The production method features low cost, high versatility and is easily up-scalable.

The ZnO tetrapods were then compounded with a loading of 5.5 vol% into the A-component of a commercial silicone rubber (a two-component RTV, Sylgard^®^ 184 Silicone Elastomer Kit, Dow Corning) using a three-roll-mill (Torrey Hills Technologies, USA). Each sample was milled 5 times at room temperature at 80 rpm, using a 100–200 µm roll nip gap. The size of the added tetrapods ranged from 10 to 100 µm.

The procedure provides large amounts of the material (Fig. S1), with a good dispersion of the tetrapods in the matrix. To check that the mixing procedure did not destroy the tetrapods, we dissolved part of the polymer in xylene, the SEM image showing the tetrapods still present in the polymer (Fig. 1b). The tetrapods are of a more complex shape with an aspect ratio very different from the SiC particles and ZnO micro varistors described previously (shown for comparison in Fig. S2). A Flacktek Speed Mixer was used to prepare the final composites, using a mixing ratio of 10:1 of the Sylgard 184 A-component (containing the ZnO tetrapods) and the B-component, respectively. The mixing speed was 3500 rpm for 2 × 1 min at 23 °C. The slurry was then degassed in a vacuum oven, at 23 °C, for 5 min. After mixing, disk samples with a diameter of 140 mm and a thickness of 0.5 mm were prepared by compression moulding (130 °C at 10 MPa pressure for 15 min in a Servitec Laboratory Press Polystat 300S). The ZnO loading of 5 vol% in the cured silicone rubber was selected to ensure a continuous percolation network while minimizing the amount of additive in the polymer (below percolation, a conventional electrically insulating material would be obtained). The conductivity measurements were performed in a climate chamber, in which the temperature was controlled (CTS C-20/350). The high voltage electrode, made of stainless steel, was connected to a Heinziger PNC5 20,000 high voltage DC power supply. On the low-voltage side of the sample, a guarded electrode was connected to a Keithley 6512 Electrometer to measure the current. The measuring electrode was surrounded by a grounded guard electrode to prevent leakage currents. Both the measurement electrode (diameter of 20 mm) and the guard were made of brass. The test cell was electrically shielded to minimize the external noise. The polarization stage process took place under constant DC electric field for 2 h, followed by a depolarization stage at zero external electric field, when both the measurement and HV electrodes were connected to ground. The conductivity was calculated based on the 2-h measurements of the polarization (*i*_*pol*_) and depolarization (*i*_*depol*_) currents.

## Results and discussion

**Figure 2:**
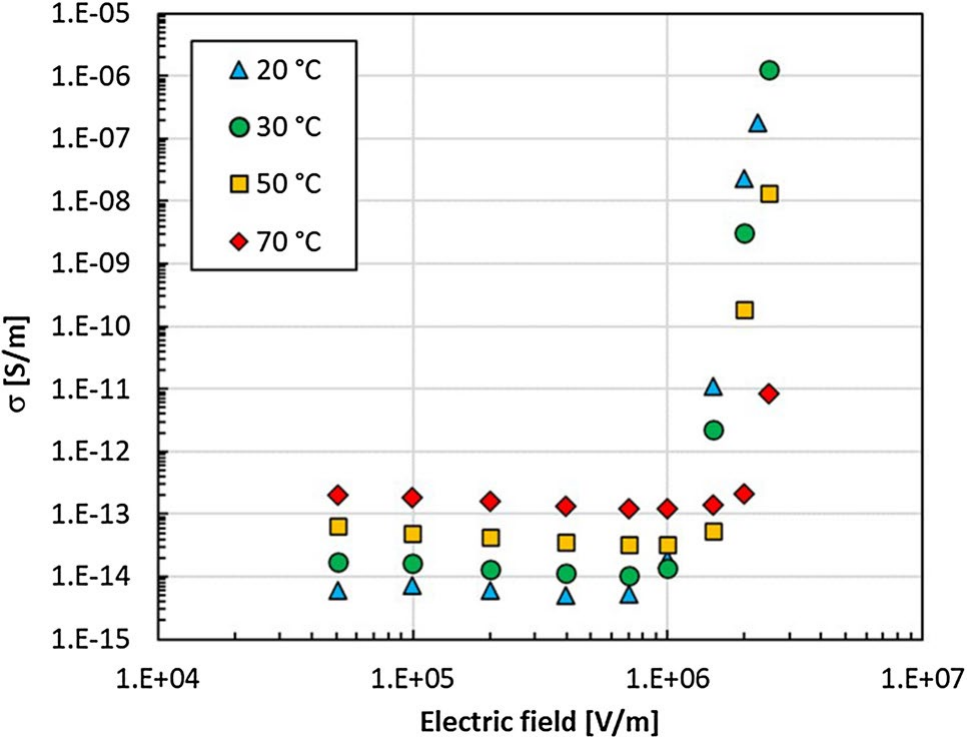
DC conductivity versus electric field at 20–70 °C for ZnO tetrapods in silicone rubber.

We measured the field-dependent DC conductivity of the silicone rubber filled with a fully percolated network (5 vol%) of ZnO tetrapods between 0.2 and 2.5 kV mm^−1^ at temperatures ranging from 20 to 70 °C (Fig. 2), a typical temperature range for RFGMs used in high voltage cable systems.

**Figure 3:**
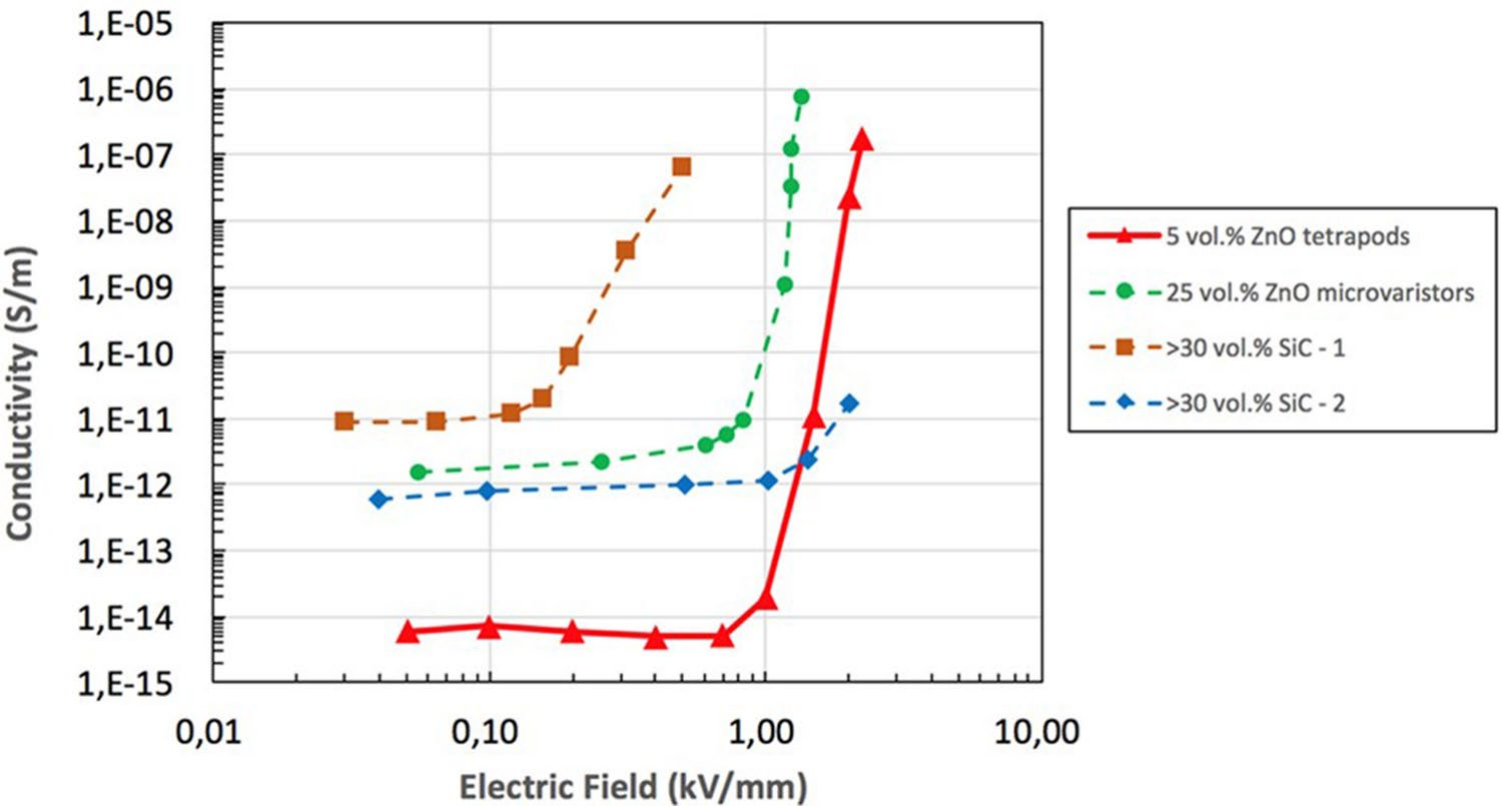
Comparison between different field grading materials at ambient temperature with typical loadings used to achieve RFG: 5 vol% ZnO tetrapods in silicone rubber compared to ZnO micro varistors and to two different SiC rubber composites, used for cable termination applications (SiC-1) and for cable joints (SiC-2) respectively^19^.

Such measurements on ZnO composites can be tricky because repeated measurements on the same sample may show quite different results^17^. The nonlinear conductivity test was reproducible when measuring one sample at three different positions, as well as at the same position on other samples of the same composition, see Fig. S3. The silicone rubber filled with a percolated network of ZnO tetrapods showed a low conductivity (*σ* ≈ 10^−14^ S m^−1^ at 20 °C) at fields below 1 kV mm^−1^. The conductivity increased with temperature, reaching 1.0–1.2 · 10^−13^ S m^−1^ at 70 °C. Above a threshold electric field *E*_*th*_, the conductivity increased rapidly. Such behaviour can be described by Eq. (1):

1 $$I = I_{0} \left( {\frac{U}{{U_{0} }}} \right)^{\alpha }$$

where *I*_0_ and *U*_0_ are, respectively, the measured current and the applied voltage in the low-conducting region and *α* is the nonlinearity coefficient. The threshold electric field *E*_*th*_ defining the transition from low to high conductivity increased linearly with temperature (Fig. S4 and Table S1), which can be explained through the interplay between polarization and conduction in the material, as discussed below. Above *E*_*th*_, the materials showed a high nonlinearity coefficient, ranging between 15 and 28, similar to that of standard RFGM materials containing ZnO micro varistors^18^. The ZnO tetrapod-silicone composite was compared with typical standard RFGM based on SiC particles or ZnO micro varistors (Fig. 3) showing higher threshold voltage, lower conductivity in the low field region (< 1 kV mm^−1^) with a larger non-linearity coefficient. Noteworthy was the excellent RFG performance observed at loadings of 5 vol%, much lower than those of typical standard composites (≥ 25 vol%).

**Figure 4:**
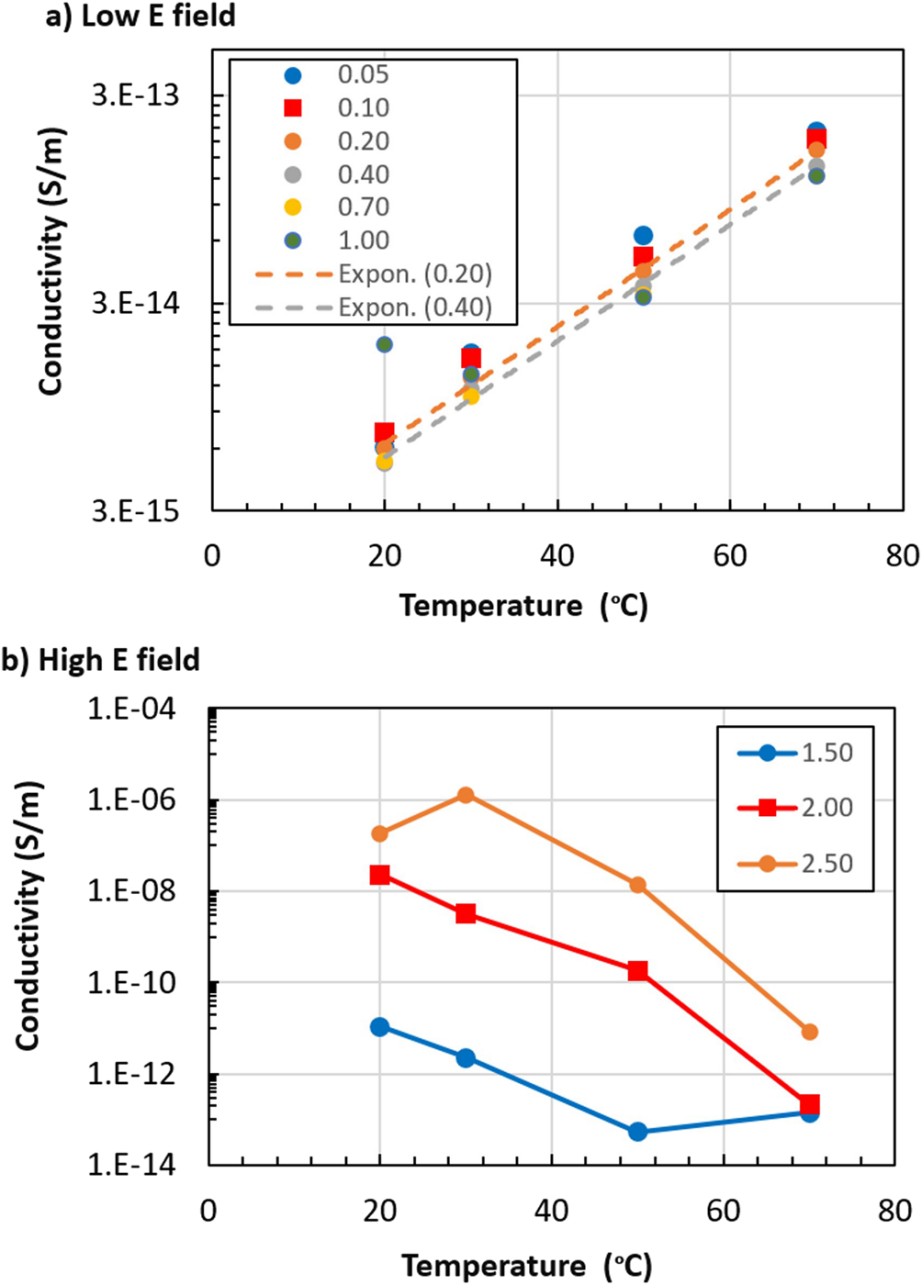
(**a**) Increase in conductivity with temperature, observed at E_th_ < 1 kV mm^−1^. (**b**) Decrease in conductivity with temperature, observed at E_th_ > 1 kV mm^−1^. Note the different Y scale between (**a**) and (**b**). The legend shows the electric field applied to perform each test, in kV mm^−1^. Dashed lines in (**a**) are exponential fitting curves of the experimental data obtained using Eq. (2). For simplicity, only two fits of the tests performed at 0.20 and 0.40 kV mm^−1^ are shown; the other fit curves look very similar to the ones shown. All fitting results are in Table S2.

The better performance could be ascribed to: (1) the high aspect ratio of the tetrapods, giving a percolated network at a low filler content^18,20^, and (2) the local field enhancement at the tips, which is able to initiate an avalanche of charge carriers. To better understand the charge transport mechanism, the temperature dependence of conductivity $\sigma$ was studied using the Arrhenius equation:

2 $$\sigma = \sigma_{0} e^{\alpha }$$

where $E_{a\sigma }$ is the activation energy of the transport and $\sigma_{0}$ is a constant. The conductivity showed a significant temperature dependence in both low and high electric field regions, but with opposite trends (Fig. 4); *σ* increased with temperature at low electric fields (*E* < *E*_*th*_) but decreased with temperature at high electric fields (*E* > *E*_*th*_).

All the measurements at low electric fields (E < 1.0 kV mm^−1^) could be fitted nicely using Eq. (2) (R^2^ = 0.99–1) but were more scattered at higher fields. Results of the analysis are reported in Table S2 in the Supplementary Information. At low fields the activation energy was 0.5–0.6 eV, while in the highest electric field region the activation energies became negative, going down to − 3.0 eV (Fig. S5). To understand this discrepancy, we can discriminate between two different types of charges contributing to this process:

1. Mobile charges effectively travelling across the material during the polarization stage giving a net DC current under the applied voltage (*Q*_*DC*_), and
2. Charges due to polarization of the material (*Q*_*pol*_), which give an equal current of opposite sign, similar to the discharging of a capacitor, during the depolarization stage (*Q*_*depol*_).

**Figure 5:**
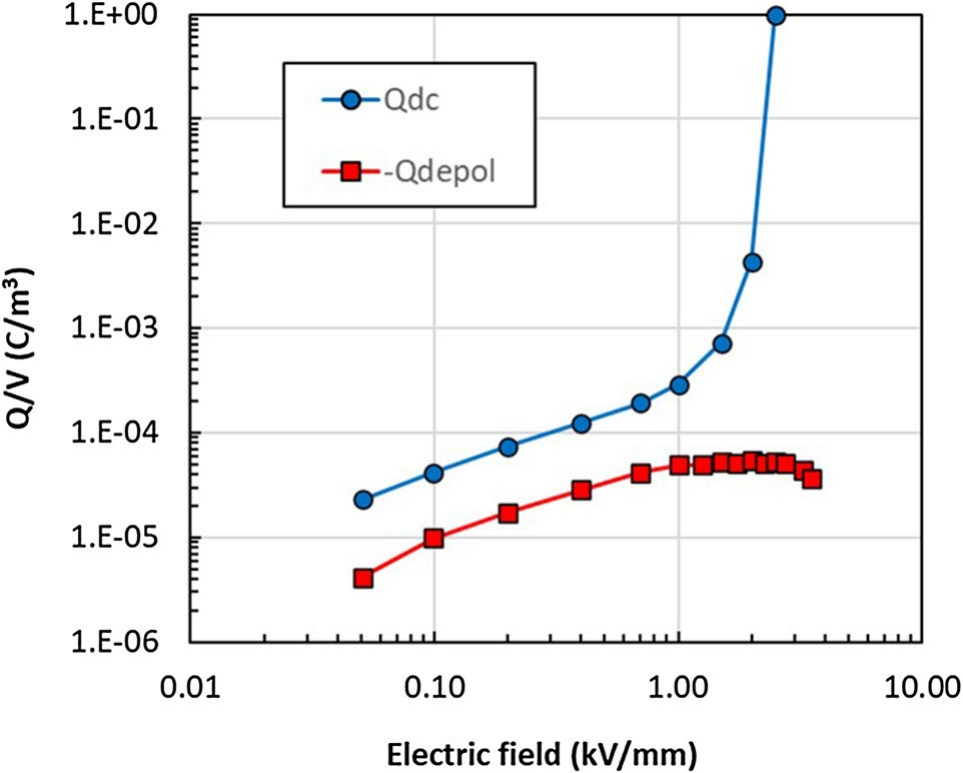
Concentration of mobile charges (Q_dc_/V) and polarizing charges (Q_depol_/V) versus electric field at 70 °C.

The effective concentration of charges measured during the charging state (*Q*_*charging*_*/V*) is defined by:

3 $$\frac{{Q_{charging} \left( {E,T} \right)}}{V} = \frac{1}{V \times 7200}\mathop \smallint \limits_{t = 0}^{7200} i_{pol} \left( {t,E,T} \right)dt$$

where *t* is the measurement time (7200 s). During the charging stage, the total charges measured are the sum of the two processes, so we have *Q*_*charging*_ = *Q*_*DC*_ + *Q*_*pol*_*, whereas* during discharge, only the current *Q*_*depol*_ is measured:

4 $$\frac{{Q_{depol} \left( {E,T} \right)}}{V} = \frac{1}{V \times 7200}\mathop \smallint \limits_{t = 0}^{7200} i_{depol} \left( {t,E,T} \right)dt$$

given that *Q*_*pol*_ = *Q*_*depol*_, we can calculate *Q*_*DC*_ as *Q*_*DC*_ = *Q*_*charging*_ − *Q*_*pol*_*.* Figure 5 shows the density of polarizing and mobile charges in the material volume at increasing electric fields, at 70 °C. *Q*_*depol*_ and *Q*_*dc*_ are comparable at low electric fields (0.05–0.7 kV mm^−1^), but at fields ≥ 1 kV mm^−1^, *Q*_*depol*_ saturates, while *Q*_*dc*_ increases significantly. A similar behaviour was observed for all temperatures tested. Figure S5 and Table S3 shows that, while the activation energy of polarizing charges (*E*_*pol*_) was nearly constant over all electrical fields, the activation energy of the effective DC charges (*Q*_*dc*_) became negative beyond the threshold electric field, *E*_*th*_.

**Figure 6:**
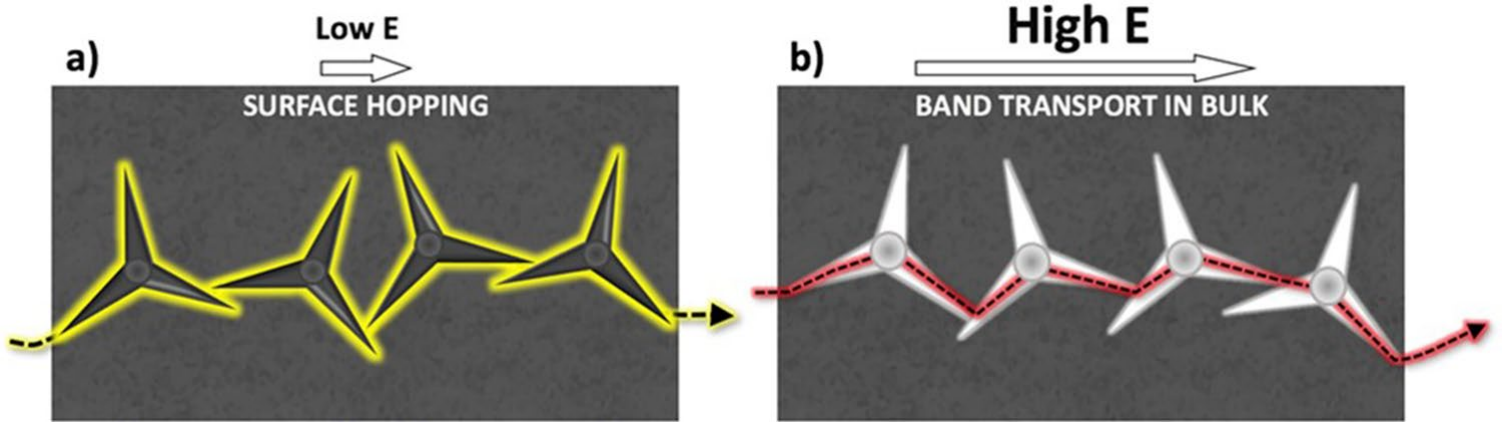
Schematic representation of (**a**) charge transport mechanism at low electric fields, due to thermally activated hopping, and (**b**) charge transport mechanism at high electric fields, due to band transport in ZnO.

Thermally activated hopping of charge carriers across spatially, as well as energetically, localized states has been widely reported to be a primary transport mechanism at low electric field in many amorphous semiconductors, dielectrics or composites such as SiC nanoparticles in silicone rubber^20,21^. In the SiC composite, charge transport was due to thermally activated hopping of holes, occurring through thin rubbery layers between the SiC particles exhibiting an activation energy ≈ 0.4–0.6 eV. This is in good agreement with the increase in conductivity (Fig. 4a) and with the positive activation energies of conduction measured here for low electric fields (*E*_*aσ*_ ranging between 0.59 and 0.47 eV, see Table S3). The measured conductivity (≈ 1·10^−14^ S m^−1^) was higher than that of pure silicone rubber (3·10^−16^ S m^−1^), also indicating the presence of some enhancing conduction mechanism. All these results indicate that, at low electric fields, conduction in the tetrapod-SiR system is due to thermally activated hopping, taking place at the silicone–ZnO interface (See Fig. 4a and Mechanism in Fig. 6a). At higher electric fields, on the other hand, the drastic change from positive to negative activation energies for the mobile charge at *E*_*th*_ indicates that the mobile charges are easily formed, as if entering a broad band of states where the charges are delocalized, with the high electric field allowing electrons to be directly injected into the material. The conductivity decreased with temperature in the investigated range 20–70 °C, for example from 1·10^−7^ S m^−1^ (20 °C) to 8·10^−12^ S m^−1^ (70 °C) at 2.5 kV mm^−1^ (Fig. 4b).

Electrically conducting polymer composites also exhibit decreased conductivity with increasing temperature. However, in the case of such polymers this trend is also observed at low electric fields, which is not the case here^22^.

At high electric field, however, charge transport cannot be described by the Arrhenius law and can no longer be described as thermally activated hopping. The experimental data can instead be interpreted as deriving from band-like transport, typical of metals and semiconductors, occurring in the semiconducting ZnO tetrapods (Fig. 6b). At high electric fields, charges can cross the 3.37 eV bandgap of ZnO, thereby creating the sharp increase in *Q*_*DC*_ charges observed in Fig. 5. For semiconductors, where lattice scattering (phonon scattering) is a dominating mechanism, the mobility decreases with increased temperature according to Sze and Lee^23^, and this explains the trend observed experimentally (Fig. 4b).

The presence of two types of charges could also explain the observed increase in E_th_ with temperature (Fig. S4). Q_depol_/V saturates at high fields, while Q_dc_/V continues to increase (Fig. 5). Also, Q_depol_/V increases linearly with temperature, and the linear dependence is observed also for E_th_ suggesting that the threshold voltage depends on the complete filling of the Q_depol_ states. When all Q_depol_ states are filled, a change in transport regime occurs and the conduction increases significantly. The anisotropic, spiked shape of the ZnO tetrapods helps to create an inhomogeneous distribution of charges in the material, triggering this process and giving a similar transition in charge transport compared to conventional ZnO varistors, while maintaining a lower leakage current in the low field region. This is beneficial for HVDC field grading applications because it can minimize the resistive loss during continuous use at these lower fields.

Finally, we chose in this work to focus on the study of the charge transport mechanism, rather than performing a classical conductivity/concentration study to detect percolation thresholds, because this material has never been tested before for RFGM. Giving the high technological importance of the material and its potential applications, we intend to study percolation onset behaviour as already carried out for in similar systems^10,12,14,15^.

## Conclusions

Resistive Field Grading Materials (RFGMs) are used in highly electrically stressed regions in electrical insulation systems of high-voltage direct-current (HVDV) cable systems. Here, we have shown that silicone rubber, filled with 5 vol% ZnO tetrapods, can be used as a new type of RFGM, reducing localised electric fields at over-voltages, and thus the electrical stress at critical positions in HVDC insulation, while minimizing resistive loss during continuous use at low fields. We observed an increase in the electrical conductivity of eight orders of magnitude (from ≈ 10^−14^ S m^−1^ to ≈ 10^−6^ S m^−1^) when the electric field was increased from 0.05 to 2.5 kV mm^−1^, with a nonlinearity coefficient (*α*) ranging between 15 and 28. The conductivity showed a strong temperature dependence in both low and high electric field regions. In the low electric field region (0.05–0.7 kV mm^−1^), the activation energies were 0.5–0.6 eV. In the highest electric field region (1.5–2.5 kV mm^−1^) the activation energies were negative, in the range − 0.7 to − 3.0 eV. Analysis of the experimental data indicates that charge transport in the low electric field region was dominated by thermally activated hopping of charge carriers across spatially, as well as energetically, localized states in an interfacial layer of silicone rubber between the ZnO tetrapods. At higher electric fields the activation energy had a negative temperature dependence, due to band charge transport occurring along the ZnO semiconducting tetrapods.

The approach described here is novel, robust, and allows over-voltage protection in high voltage components with a significantly lower degree of filling (5 vol%) compared to conventional resistive field grading composites. This leads to several advantages, the most important one being the reduced leakage current at operational electric fields which are of great interest for industrial applications in high-voltage direct current cable insulation systems, where such RFGMs are required.

## Supplementary Information

Supplementary Information.
