# Supplementary material for: Tuneable conductivity at extreme electric fields in ZnO tetrapod-silicone composites for high-voltage power cable insulation
Source: Sci Rep. 2022 Apr 11;12:6035. doi: 10.1038/s41598-022-09966-4 (PMC9001661; doi:10.1038/s41598-022-09966-4)
Supplement: Supplementary file 1 — Supplementary Information. [file 41598_2022_9966_MOESM1_ESM.docx]

SUPPLEMENTARY INFORMATION

Tuneable conductivity at extreme electric fields in ZnO tetrapods-silicone composites for high-voltage power cable insulation

Helena Greijer, Nicola Mirotta, Emanuele Treossi, Filippo Valorosi, Fabian Schütt, Leonard Siebert, Yogendra Kumar Mishra, Rainer Adelung, Vincenzo Palermo,* Henrik Hillborg*


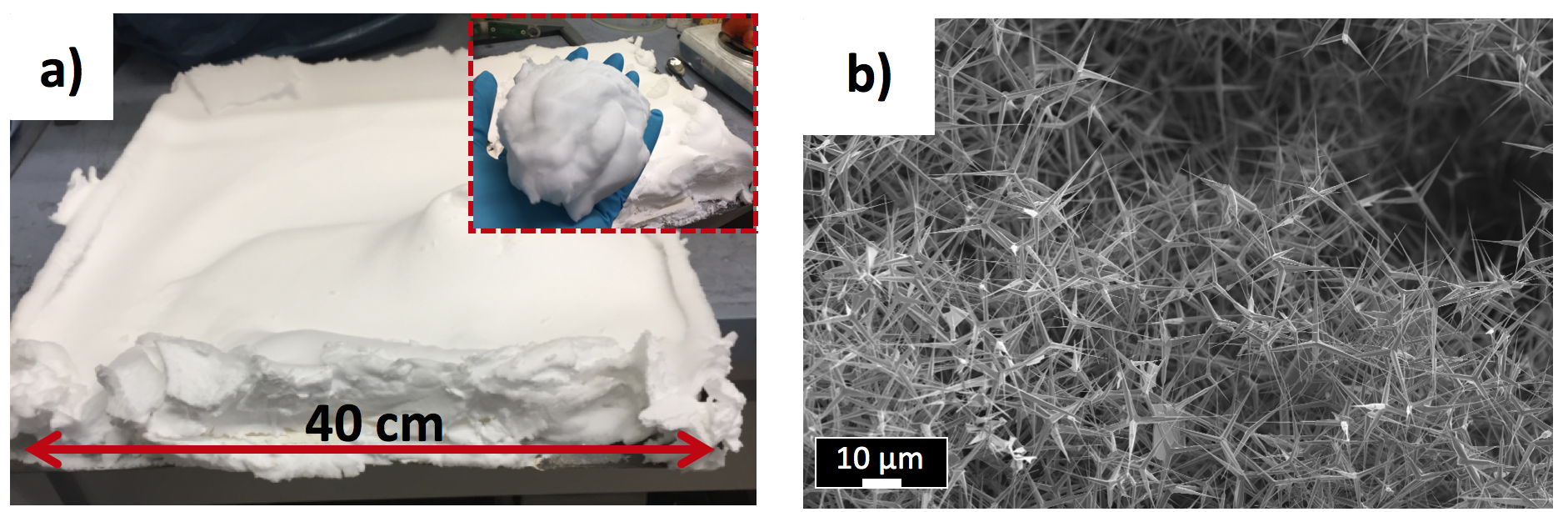


Figure S1. a) Silicone rubber filled with 5 vol.% ZnO tetrapods, and b) corresponding SEM image.


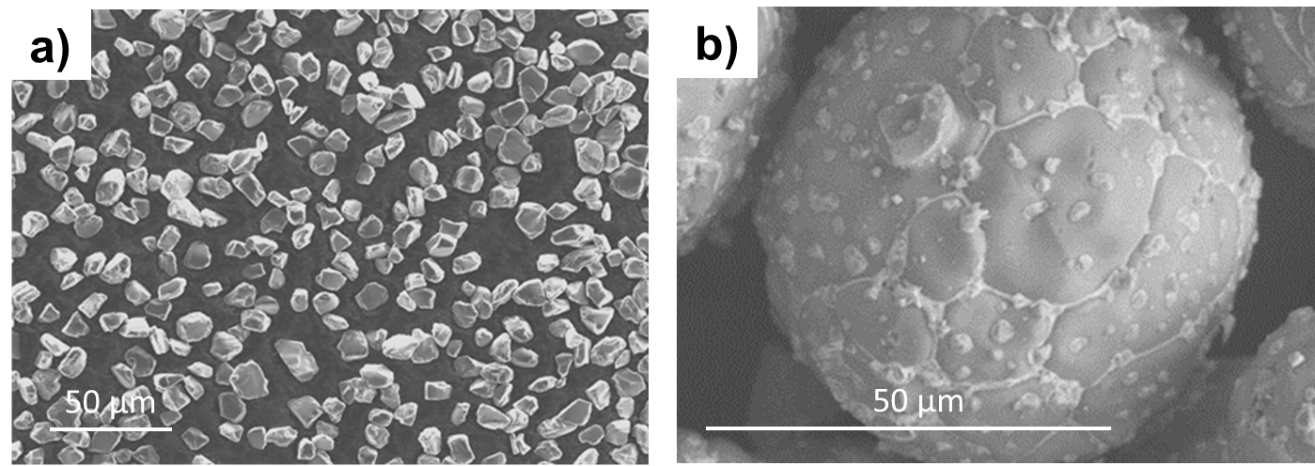


Figure S2. a) SEM micrographs of a) SiC particles, and b) ZnO microvaristors.


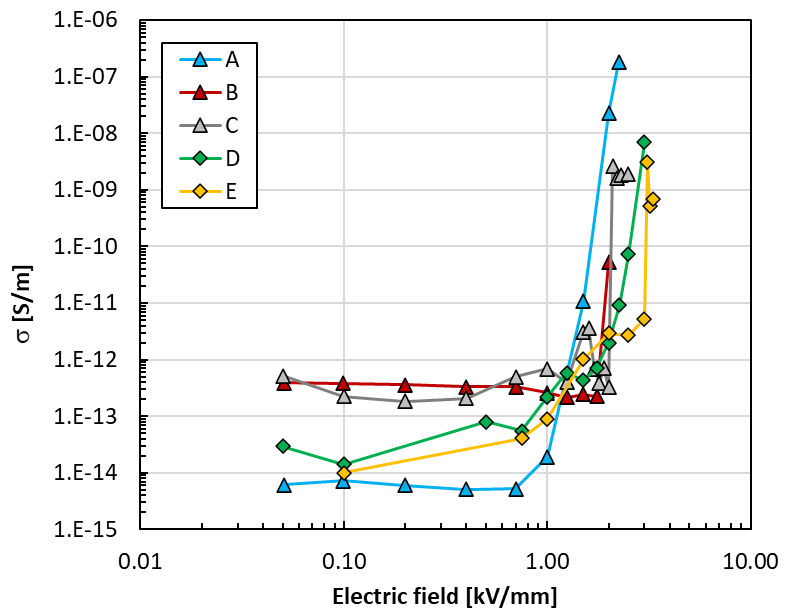


Figure S3. Reproducibility of DC conductivity vs. electric field at 20˚C for 5 vol.% ZnO tetrapods in silicone rubber. The measurements were performed at three different positions on the same sample (A-C), as well as measurements repeated on other samples (D-E). The data show some variation of resistivity at low fields, ranging from 10^-12^-10^-14^ S-m^-1^ according to the measurement; such variability is normal and can be expected for conductivity measurements of highly insulating materials. The important figure of merit, the threshold value of the electric field, is however highly reproducible, and was measured in all cases within a narrow range (1-3 kV/mm).


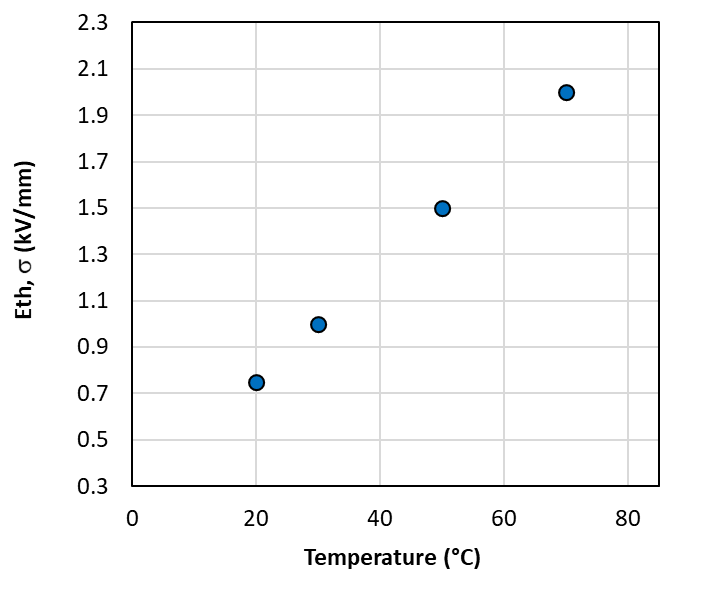


Figure S4. The threshold electric field (*E_th_*) vs. temperature.


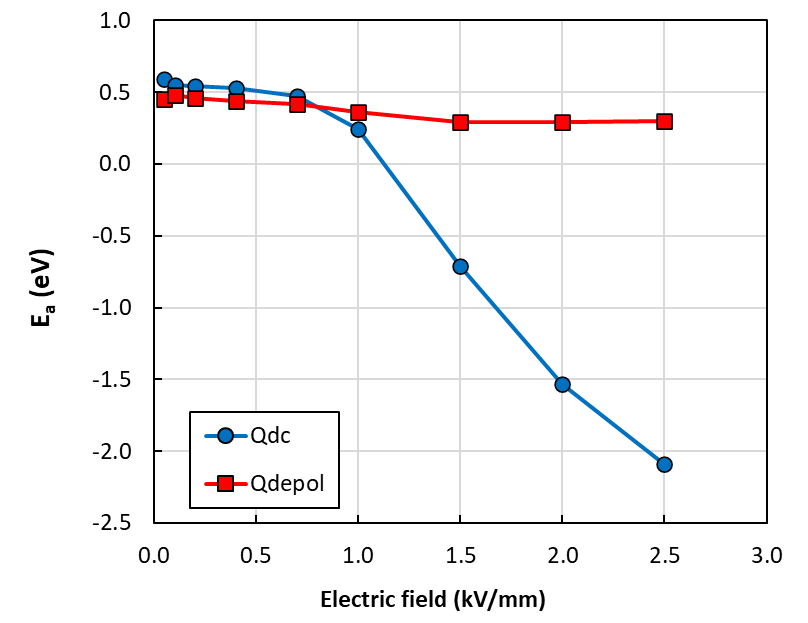


Figure S5. The activation energy of mobile (*E_aQdc_*) and polarization (*E_aQdepo_*_l_) charge formation obtained from fitting of the Arrhenius equation.

Table S1. Threshold electric field for conduction and non-linearity coefficient vs. temperature.

| **Temp. (˚C)** | **E_th, σ_ (kV/mm)** | **α** |
| --- | --- | --- |
| 20 | 0.75 | 15 |
| 30 | 1.0 | 21 |
| 50 | 1.5 | 28 |
| 70 | 2.0 | 25 |

Table S2. Activation energy of conduction for different electric fields.

| **E (kV/mm)** | **E_aσ_ (eV)** | **No. of data points** | **Model fit (R^2^)** |
| --- | --- | --- | --- |
| 0.05 | 0.59 | 4 | 0.99 |
| 0.1 | 0.55 | 4 | 1.00 |
| 0.2 | 0.56 | 4 | 1.00 |
| 0.4 | 0.56 | 4 | 1.00 |
| 0.7 | 0.54 | 4 | 1.00 |
| 1.0 | 0.34 | 4 | 0.82 |
| 1.5 | -0.72 | 5 | 0.73 |
| 2 | -2.03 | 5 | 0.96 |
| 2.5 | -2.95 | 4 | 0.96 |

*Table S3. The calculated activation energy of mobile (E_aQdc_) and polarizing (E_aQdepol_) charge formation and the quality of the model fits.*

| **E (kV/mm)** | **E_aQdc_ (eV)** | **Model fit (R^2^)** | **E_aQdepol_ (eV)** | **Model fit (R^2^)** |
| --- | --- | --- | --- | --- |
| 0.05 | 0.59 | 0.99 | 0.45 | 0.96 |
| 0.1 | 0.55 | 1.00 | 0.48 | 0.94 |
| 0.2 | 0.54 | 1.00 | 0.46 | 0.93 |
| 0.4 | 0.53 | 1.00 | 0.44 | 0.95 |
| 0.7 | 0.47 | 1.00 | 0.42 | 0.95 |
| 1.0 | 0.24 | 0.82 | 0.36 | 0.85 |
| 1.5 | -0.71 | 0.73 | 0.29 | 0.76 |
| 2 | -1.53 | 0.96 | 0.29 | 0.88 |
| 2.5 | -2.09 | 0.96 | 0.30 | 0.98 |
